# Deep Eutectic Solvents as Effective Reaction Media for the Synthesis of 2-Hydroxyphenylbenzimidazole-Based Scaffolds en Route to Donepezil-Like Compounds

**DOI:** 10.3390/molecules25030574

**Published:** 2020-01-28

**Authors:** Luca Piemontese, Roberta Sergio, Federica Rinaldo, Leonardo Brunetti, Filippo M. Perna, M. Amélia Santos, Vito Capriati

**Affiliations:** 1 Dipartimento di Farmacia–Scienze del Farmaco, Università degli Studi di Bari «Aldo Moro», Via E. Orabona 4, I-70125 Bari, Italy; r.sergio2@studenti.uniba.it (R.S.); f.rinaldo@studenti.uniba.it (F.R.); l.brunetti2@studenti.uniba.it (L.B.); filippo.perna@uniba.it (F.M.P.); 2 Consorzio C.I.N.M.P.I.S., Via E. Orabona 4, I-70125 Bari, Italy; 3 Centro de Química Estrutural, Instituto Superior Técnico, Universidade de Lisboa, Av. Rovisco Pais 1, 1049-001 Lisboa, Portugal; masantos@ist.utl.pt

**Keywords:** deep eutectic solvents, 2-hydroxyphenylbenzimidazole, Alzheimer’s disease

## Abstract

An unsubstituted 2-hydroxyphenylbenzimidazole has recently been included as a scaffold in a series of hybrids (including the hit compound PZ1) based on the framework of the acetylcholinesterase (AChE) inhibitor Donepezil, which is a new promising multi-target ligand in Alzheimer’s disease (AD) treatment. Building upon these findings, we have now designed and completed the whole synthesis of PZ1 in the so-called deep eutectic solvents (DESs), which have emerged as an unconventional class of bio-renewable reaction media in green synthesis. Under optimized reaction conditions, the preparation of a series of 2-hydroxyphenylbenzimidazole-based nuclei has also been perfected in DESs, and comparison with other routes which employ toxic and volatile organic solvents (VOCs) provided. The functionalization of the aromatic ring can have implications on some important biological properties of the described derivatives and will be the subject of future studies of structure-activity relationships (SARs).

## 1. Introduction

Alzheimer’s disease (AD) is recognized as a social and economic problem with an annual incidence of 34/1000 persons over 60 years old [1,2,3,4]. It is estimated that, in the absence of effective therapies, the number of people with dementia will reach more than 130 million worldwide by 2050 [5]. Although numerous clinical trials have been projected and realized [5,6,7], only five symptomatic drugs have been approved to date for the use as anti-AD agents at international level. Tacrine, which represented the first breakthrough in the AD therapy, has been discontinued from the market in several countries because of its severe adverse events [8].

Considering the specific pathogenesis of AD, a new generation of ligands has recently been explored as multifunctional molecules aimed at simultaneously acting on two or more disease features (multi-target directed ligands) so as to achieve synergistic or at least complementary therapeutic effects. The model based on the “one molecule/multiple targets” concept has led to the design of novel molecules frequently inspired by natural products or bio-active synthetic molecules [2]. The most explored strategy for multi-target anti-AD drugs is based on the “cholinergic hypothesis”. This involves the repositioning of drugs already used in therapy such as tacrine, donepezil, memantine, rivastigmine [2,9,10,11] as starting molecules to hit other AD targets [12]. According to the so-called “metal hypothesis”, several metal-chelating moieties have been incorporated in these structures [5,7,8]. Indeed, the biometal dyshomeostasis (copper and zinc cations but also iron and aluminum) in AD is known to be involved in Aβ aggregation [13,14,15]. Moreover, the redox activity of these ions may lead to the formation of reactive oxygen species (ROS), which are known to play an important role in chronic inflammation eventually responsible for the oxidative stress of neuronal cells [2,6,16,17,18].

In the last years, our research groups have synthesized and assayed new multi-functional ligands with chelating abilities towards Cu^2+^ and Zn^2+^, potentially useful in the AD treatment based on the framework of the acetylcholinesterase (AChE) inhibitor Donepezil and 2-hydroxyphenylbenzimidazole [19,20,21]. Benzimidazole-based nuclei have been the subject of intense investigation in recent years as they proved to be important scaffolds for the preparation of other bioactive compounds [22,23,24,25]. For example, they have been included in several drugs and drug-like candidates such as the antihypertensive drug Telmisartan and the antiviral drug Maribavir, in several anti-inflammatory and antiulcer agents like the blockbuster drug Omeprazole [22,23], and in various β-secretase (BACE1) inhibitors and other AD targets [25,26,27].

Because of stringent environmental legislation to address the climate crisis, urgent actions are needed to be taken in the chemical production, in particular, to progressively replace extensively used conventional and often hazardous volatile organic compounds (VOCs), which are known to contribute to over 80% of the organic waste produced, in favor of safer and more environmentally responsible solvents. In this context, the so-called deep eutectic solvents (DESs) have emerged as a novel promising class of green solvents as they are non-flammable, highly thermally stable, with practically no vapor pressure, and thus low volatility [28]. They are combination of two or three safe and cheap components (Lewis or Bronsted acids and bases, which can contain a variety of anionic and/or cationic species) that form, through hydrogen bond formation, a eutectic liquid mixture at a temperature far below than that of either of the individual components. Typical DES components come from renewable sources [e.g., choline chloride (ChCl), glycerol (gly), urea, natural carboxylic acids, amino acids, polyalcohols]. Therefore, their biodegradability is high and their toxicity is non-existent or very low. In addition, they can be easily prepared and exhibit tunable physicochemical properties [28]. Because of their ability to act also as catalysts and reagents [29,30], DESs have been primarily investigated in extraction and separation processes [31,32,33,34], in material sciences [35], for metal electrodeposition [36], and for the synthesis of heterocycles [37]. Emerging and hot fields of applications are represented by organometallics [38,39,40], metal- [41,42,43,44,45,46,47,48], bio- [49,50,51,52,53], and organo-catalysis [54,55,56,57], electrochemistry [58], photosynthesis [59] and energy technology [60,61]. Building upon our interests in the synthesis of drugs and heterocycles using eco-friendly reaction media like DESs [43,44,45,46,47,55,62,63] and water [64], herein we report the sustainable preparation of several 2-hydroxyphenylbenzimidazole derivatives and the whole synthesis of PZ1 [19] in selected eutectic mixtures.

## 2. Results and Discussion

**Figure 1:**
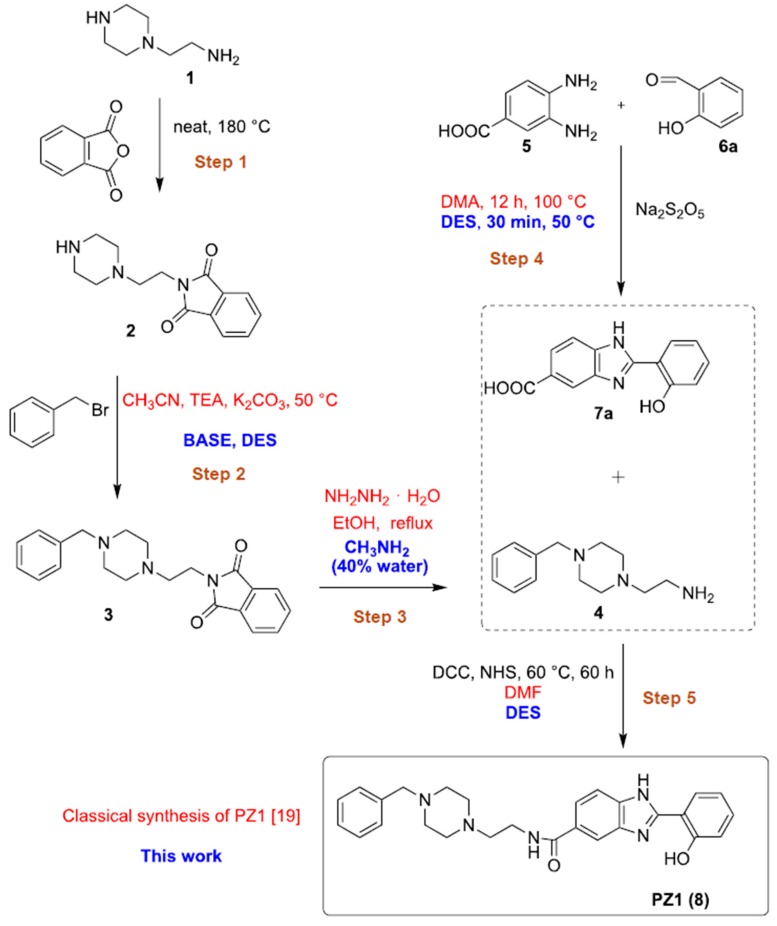
Classical (red, [19]) and new green (blue) procedure for the synthesis of PZ1.

The role played by Zn and Cu cations in the Aβ deposition and stabilization has been a matter of debate in the literature in the last decade as well as the possibility that metal chelating agents can lead to the dissolution of Aβ and/or aggregation by preventing the interaction between the metal and the protein [2,11,17,21,65,66,67,68,69]. According to the metal hypothesis of AD, it is important the inclusion of chelating moieties in the design of therapeutic/diagnostic agents [69]. Our groups recently focused on the synthesis and the biological evaluation of new Donepezil-like conjugates, and especially interesting were the results obtained from biological assays on PZ1 [19,20,21]. The synthesis of this new hit compound was designed using VOCs ([19], Figure 1, red). Particularly disappointing from an environmental viewpoint were Steps 2, 4 and 5 in which it was made use of toxic and anhydrous CH_3_CN (step 2), *N*,*N*-dimethylacetamide (DMA) (Step 4), *N*,*N*-dimethylformamide (DMF) (Step 5), and of a carcinogenic reactant like hydrazine hydrate in Step 3. In addition, Step 4 also required up to 12 h reaction time for completion. Thus, we decided to reshape the whole synthesis of PZ1 using DESs as environmentally responsible reaction media (Figure 1, blue). 

The selective protection of the primary amine moiety of **1**, performed by reacting **1** with phthalic acid anhydride at 180 °C for 5 h under solventless conditions, delivered adduct **2** in quantitative yield (>98% yield) [19,70] (Figure 1, Step 1). The benzylation of the secondary amine of the piperazine moiety of **2** en route to adduct **3** (Figure 1, Step 2) was originally carried out in acetonitrile and in the presence of a couple of bases, used in excess (64% yield) (Table 1, entry 1). An extensive screening of bases [KOH, *t*-BuOK, K_2_CO_3_, triethylamine (TEA)] in different hydrophilic [28,71,72] and hydrophobic [73] eutectic mixtures as solvents, at a temperature of 50 or 100 °C (Table 1), revealed that TEA (2 equiv) either in ChCl/propylene glycol (PG) (1:3 mol mol^−1^) or in Bu_4_NCl/gly (1:4 mol mol^−1^) [72] were the best combinations as they delivered **3** in 64–68% yield (Table 1, entries 15,19). 

The deprotection of the phthalimido moiety of **3** (Figure 1, Step 3) was realized using MeNH_2_ (40% aq. solution) in place of a carcinogenic reactant such as hydrazine hydrate [19,74]. In this way, the *N*-benzylated adduct **4** was isolated in 95% yield. A similar yield (95%) was observed by reacting **2** with MeNH_2_ in a ChCl/gly (1:2) eutectic mixture with 40 w% water. On the other hand, by alternatively using the eutectic mixture ChCl/PG (1:3) + 40 w%, adduct **4** was obtained in 45% yield only. The synthesis of 2-hydroxyphenylbenzimidazole **7a**, via a cyclodehydration reaction between 3,4-diaminobenzoic acid (**5**) and salicylaldehyde (**6a**) (Figure 1, Step 4), was also optimized in DESs using Na_2_S_2_O_5_ as the oxidant (Table 2). We screened three prototypical ChCl-based eutectic mixtures whose hydrogen bond component was basic (ChCl/urea; 1:2 mol mol^−1^), neutral (ChCl/gly; 1:2 mol mol^−1^) or acidic [(ChCl-l-lactic acid (LA); 1:2 mol mol^−1^)]. All reactions were monitored through TLC analysis and stopped after complete consumption of the starting materials. As shown in Table 2, very good yields (up to 80%) were obtained in each case in short reaction times (30 min) at 100 °C (Table 2, entries 1–3). As for the ChCl/gly eutectic mixture, the percentage yield of **7a** could be increased to up to 84% running the reaction at 50 °C (Table 2, entry 4), whereas a temperature as low as 25 °C was detrimental even after 24 h reaction time (36% yield) (Table 2, entry 5). By changing the oxidant from Na_2_S_2_O_5_ to the commercially available urea-hydrogen peroxide or by alternatively running the cyclodehydration reaction under air in the absence of any additional oxidant reagent, the yield of **7a** dropped down to 17% and 29% (^1^H NMR analysis), respectively, the remaining being a complex mixture of unidentified products (Table 2, entries 6,7). Compound **7a** was found to precipitate directly from the above ChCl/gly mixture after dilution with water. Thus, it was isolated by simple filtration on a Büchner funnel and washing with a few drops of CH_2_Cl_2_. The same reaction, run in DMA at 100 °C, in the presence of Na_2_S_2_O_5_, provided **7a** in 67% yield only after 12 h reaction time (Table 2, entry 8).

**Figure 2:**
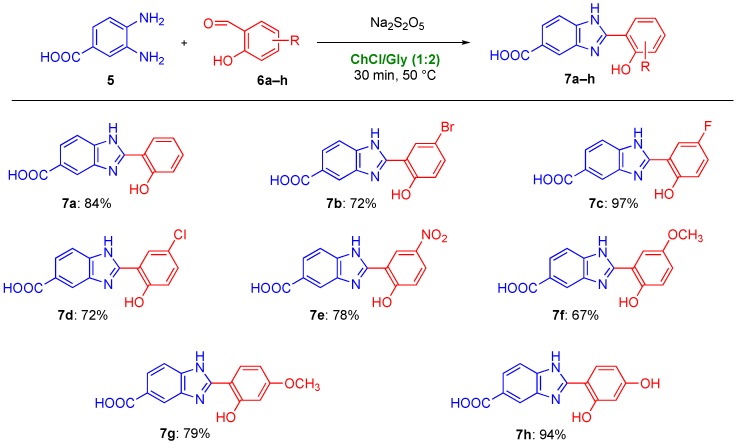
Scope of the cyclodehydration reaction for the synthesis of 2-hydroxyphenylbenzimidazole derivatives **7**. Yields refer to isolated products.

Possibly looking forward to developing and testing novel donepezil-hybrids, we decided to broaden already at this stage the scope of the aforementioned cyclodehydration reaction using ChCl/gly as a privileged reaction medium. The functionalization of the phenolic acid component may indeed contribute to modify the biological properties of the corresponding adducts, thereby tuning their chelating properties for the treatment of AD. In line with this strategy, Liang et al. recently synthesized a series of novel halogenated 8-hydroxyquinolines as derivatives of Clioquinol (CQ), which is a well-known prototypical metal-chelating drug [68]. CQ was studied up to phase II clinical trial with promising results, but it was later discontinued because of issues encountered during the development of the industrial production process [2,75]. In particular, it was noticed that the introduction in the CQ structure of powerful electron-withdrawing groups led to an improved metal-chelating activity [68]. Another research group recently explored also the potentiality of these substituted nuclei as antioxidant agents [76]. To our delight, by reacting a variety of salicylaldehyde derivatives **6b**–**h**, decorated with electron-donating and electron-withdrawing substituents, with **5**, the desired 2-hydroxyphenylbenzimidazole derivatives **7b**–**h** could be smoothly synthesized in 72–97% yield within 30 min reaction tim at 50 °C (Figure 2). Of note, using DMA as the reaction medium, the above adducts have been reported to be prepared in 51–74% yield after 12 h reaction time at 100 °C [77]. The synthesis of 1,2-disubstituted or 2-substituted benzimidazoles has also been recently achieved using *o*-phenylenediamine (*o*-PDA) both as a component of the eutectic mixture ChCl/*o*-PDA (1:1 mol mol^−1^) and as a reagent in combination with different aldehydes [78]. 

Finally, the one-pot two-step synthesis of PZ1 (**8**) in DESs (Figure 1, Step 5) was investigated by reacting the *N*-benzylated adduct **4** (Figure 1, Step 3) with 2-hydroxyphenylbenzimidazole derivative **7a** (Figure 1, Step 4) (Table 3). The preparation of PZ1 from **4** and **7a** has only been performed to date in DMF, working at 25 °C for 60 h, with the product isolated in 21% yield (Table 3, entry 1). The use of ChCl/PG (1:3) as the solvent proved to be better with respect to other hydrophilic and hydrophobic eutectic mixtures as it provided PZ1 in an overall 30% yield at 60 °C after 60 h reaction time (Table 3, entries 2–8). The presence of both *N*,*N*′-dicyclohexylcarbodiimide (DCC) and *N*-hydroxysuccinimide (NHS) was also essential for the in situ formation of the amide moiety of **8** (Table 3, entries 9,10).

## 3. Materials and Methods

### 3.1. General Methods

^1^H NMR and ^13^C NMR spectra were recorded on a Bruker 600 MHz spectrometer and chemical shifts are reported in parts per million (δ). FT-IR spectra were recorded on a Perkin-Elmer 681 spectrometer. GC analyses were performed on a HP 6890 model, Series II by using a HP1 column (methyl siloxane; 30 m, 0.32 mm, 0.25 μm film thickness). Analytical thin-layer chromatography (TLC) was carried out on pre-coated 0.25 mm thick plates of Kieselgel 60 F_254_; visualization was accomplished by UV light (254 nm) or by spraying a solution of 5 % (*w*/*v*) ammonium molybdate and 0.2 % (*w*/*v*) cerium(III) sulfate in 100 mL 17.6 % (*w*/*v*) aq. sulfuric acid and heating to 473 K until blue spots appeared. Chromatography was conducted by using silica gel 60 with a particle size distribution 40–63 μm and 230–400 ASTM. GC-MS analyses were performed on HP 5995C model. High-resolution mass spectrometry (HRMS) analyses were performed using a Bruker microTOF QII mass spectrometer equipped with an electrospray ion source (ESI). Reagents and solvents, unless otherwise specified, were purchased from Sigma-Aldrich (Sigma-Aldrich, St. Louis, MO, USA) and used without any further purification.

### 3.2. Preparation of Deep Eutectic Solvents (DESs)

DESs [choline chloride (ChCl)/propylene glycol (PG) (1:3 mol mol^−1^); ChCl/l-lactic acid (LA) (1:2 mol mol^−1^); d-fructose/urea (3:2 *w*/*w*); d-fructose/ChCl (2:1 mol mol^−1^); ChCl/urea (1:2 mol mol^−1^); ChCl/glycerol (gly) (1:2 mol mol^−1^; dl-menthol/LA (1:2 mol mol^−1^); Bu_4_NBr/gly (1:4 mol mol^−1^); Bu_4_NCl/gly (1:4 mol mol^−1^)] were prepared by heating under stirring at 60–80 °C for 10–30 min the corresponding individual components until a clear solution was obtained.

### 3.3. Synthesis and Characterization Data of 2-[2-(4-benzylpiperazin-1-yl)ethyl]isoindoline-1,3-dione (**2**)

Phthalic acid anhydride (0.5 mmol) and 1-(2-aminoethyl)piperazine **1** (0.5 mmol) were heated at 180 °C for 5 h to give 2-[2-(piperazin-1-yl)ethyl]isoindoline-1,3-dione (**2**) as a dark brown solid in >98% yield (^1^H NMR analysis) [19,70].

Table 1, entry 1: the resulting dark brown solid (**2**), (0.5 mmol) was mixed with K_2_CO_3_ (3.3 mmol), triethylamine (TEA) (1.0 mmol) and benzyl bromide (1.15 mmol) and refluxed in acetonitrile (2 mL) for 3 h at 50 °C. Then, the mixture was cooled to room temperature and 5 mL of H_2_O were added. The resulting aqueous suspension was then extracted with AcOEt (3 × 10 mL). The combined organic phase were dried over anhydrous Na_2_SO_4_ and concentrated under reduced pressure. The crude product was purified by flash-chromatography (silica gel; eluent: CH_2_Cl_2_/MeOH/TEA 98:2:0.1) to give **3** as a yellow solid (64% yield).

Table 1, entry 19: the resulting dark brown solid (**2**), (0.5 mmol), TEA (1.0 mmol) and benzyl bromide (1.0 mmol) were dissolved in the Bu_4_NCl/gly eutectic mixture (1.0 g) under magnetic stirring, and the resulting mixture was then heated at 50 °C for 24 h. After this time, the reaction mixture was cooled to room temperature and 5 mL of H_2_O were added. The resulting aqueous suspension was then extracted with AcOEt (3 × 10 mL). The combined organic phases were washed with brine (10 mL), dried over anhydrous Na_2_SO_4_ and concentrated under reduced pressure. The crude product was purified by flash-chromatography (silica gel; eluent: CH_2_Cl_2_/MeOH/TEA 98:2:0.1) to give **3** as a yellow solid (68% yield).

*2-[2-(4-benzylpiperazin-1-yl)ethyl]isoindoline-1,3-dione (***3***).*^1^H NMR (400 MHz, CDCl_3_), δ (ppm): 2.39–2.52 (m, 8 H, piperazine), 2.60 (t, 2 H, *J* = 6.7 Hz, phthalimide-CH_2_CH_2_N), 3.44 (s, 2 H, NCH_2_Ph), 3.78 (t, 2 H, *J* = 6.7 Hz, phthalimide–CH_2_CH_2_N), 7.19–7.27 (m, 5 H, aromatics, Ph–CH_2_), 7.67–7.69 and 7.80–7.82 (m, 4 H, phthalimide). ^13^C NMR (150 MHz, CDCl_3_) δ (ppm): 35.3, 53.0, 53.1, 55.7, 63.0, 123.2, 127.0, 128.2, 129.2, 132.2, 133.8, 138.1, 168.3. GC-MS (*m*/*z*): 349 (M^+^, 7), 189 (100), 91 (47). HRMS [M + H]^+^: calculated 350.1863; found 350.1858.

### 3.4. Synthesis and Characterization Data of 2-(4-benzyl-1-piperazinyl)ethanamine (**4**)

Compound **3** (1.0 mmol) was dissolved in an aq. solution of MeNH_2_ (40% *w*/*w*, 3.0 mL) under magnetic stirring for 24 h at room temperature. After this time, an aq. solution of NaOH (20% *w*/*w*, 3.0 mL) was added. After 2 h, NaCl (0.3 g) was added, and the resulting solution was extracted with AcOEt (3 × 10 mL). The combined organic phases were washed with brine (10 mL), dried over anhydrous Na_2_SO_4_ and concentrated under reduced pressure to give **4** as a yellow oil (95% yield). 

^1^H NMR (400 MHz, CDCl_3_), δ (ppm): 2.35–2.43 (m, 10 H, piperazine, NH_2_–CH_2_CH_2_N), 2.73 (t, 2 H, *J* = 6.1 Hz, NH_2_–CH_2_CH_2_N), 3.46 (s, 2 H, NCH_2_Ph), 7.18–7.27 (m, 5 H, aromatics, Ph–CH_2_). ^13^C NMR (150 MHz, CDCl_3_) δ (ppm): 38.7, 53.1, 53.2, 61.1, 63.1, 127.0, 128.2, 129.2, 138.0. GC-MS (*m/z*): 219 (M^+^, 1), 189 (100), 91 (94). HRMS [M + H]^+^: calculated 220.1808; found 220.1809.

### 3.5. Synthesis and Characterization Data of 2-(2-hydroxyphenyl)-1H-benzo[d]imidazole-5-carboxylic Acid (**7a**)

Table 2, entry 8: to a solution of salicylaldehyde (0.5 mmol) in *N*,*N*-dimethylacetamide (2 mL), 3,4-diaminobenzoic acid (0.5 mmol) and Na_2_S_2_O_5_ (0.7 mmol) were progressively added under magnetic stirring. The resulting mixture was heated at 100 °C for 12 h, and then cooled to room temperature. The mixture was finally diluted with AcOEt (10 mL), washed with brine (3 × 10 mL), dried over anhydrous Na_2_SO_4_ and concentrated under reduced pressure to give **7a** as a pale brown solid (67% yield).

Table 2, entry 4: 3,4-diaminobenzoic acid (0.5 mmol), salicylaldehyde (0.5 mmol) and Na_2_S_2_O_5_ (0.7 mmol) were progressively dissolved in a ChCl/gly eutectic mixture (1.0 g), and the resulting mixture was warmed at 50 °C for 30 min. After this time, the reaction mixture was cooled to room temperature and 10 mL of H_2_O were added. This caused the precipitation of **7a** as a pale brown solid, which was isolated by filtration on a Büchner funnel and washing with a few drops of CH_2_Cl_2_ (84% yield).

*2-(2-Hydroxyphenyl)-1H-benzo[d]imidazole-5-carboxylic acid* (**7a**) ^1^H NMR (400 MHz, DMSO-d_6_) δ (ppm): 7.02–7.08 (m, 2 H), 7.41 (t, *J* = 7.4 Hz, 1 H), 7.73 (d, *J* = 7.7 Hz, 1 H), 7.91 (d, *J* = 7.9 Hz, 1 H), 8.11 (d, *J* = 8.1 Hz, 1 H), 8.24 (s, 1 H). ^13^C NMR (100 MHz, DMSO-d_6_) δ (ppm): 168.1, 158.5, 132.7, 127.2, 125.7, 124.55, 119.7, 117.7, 112.9. ESI-MS (*m/z*): 253 (M − 1), 255 (M + 1). HRMS [(M − H]^−^: calculated 253.0619; found 253.0637.

### 3.6. Synthesis and Characterization Data of 2-(2-hydroxyphenyl)-1H-benzo[d]imidazole-5-carboxylic Acid Derivatives (**7b**–**h**)

Compounds **7b**–**h** were synthesized in the ChCl/gly eutectic mixture according to the procedure described for **7a** in Section 3.5.

*2-(5-Bromo-2-hydroxyphenyl)-1H-benzo[d]imidazole-5-carboxylic acid (***7b***).* White solid, m.p. > 300 °C, 72% yield. ^1^H NMR (600 MHz, DMSO-d_6_) δ (ppm): 7.05 (d, *J* = 8.7 Hz, 1 H, BIM-H-4), 7.55 (d, *J* = 8.7 Hz, 1 H, BIM-H-5), 7.74 (d, *J* = 8.3 Hz, 1 H, BIM-H-2), 7.91 (d, *J* = 8.3 Hz, 1 H, BIM-H-3), 8.24 (s, 1 H, BIM-H-7), 8.30 (s, 1 H, BIM-H-1), 12.95 (broad s, 1 H, COOH). ^13^C NMR (150 MHz, DMSO-d_6_) δ (ppm): 110.8, 114.9, 115.0, 120.0, 124.8, 124.9, 126.0, 129.5, 135.1, 152.4, 152.5, 157.5, 168.0. HRMS [M − H]^−^: calculated 330.9724; found 330.9722.

*2-(5-Fluoro-2-hydroxyphenyl)-1H-benzo[d]imidazole-5-carboxylic acid (***7c***).* White solid, m.p. 250 °C, 97% yield. ^1^H NMR (600 MHz, DMSO-d_6_) δ (ppm): 7.04–7.06 (m, 1 H), 7.22–7.25 (m, 1 H), 7.70–7.72 (m, 1 H), 7.88–7.90 (m, 2 H), 7.98–8.01 (m, 1 H), 8.25 (broad s, 1 H), 12.78 (broad s, 1 H). ^13^C NMR (150 MHz, DMSO-d_6_) δ (ppm): 112.8 (d, ^2^*J*_C − F_ = 25.0 Hz), 113.3 (d, ^3^*J*_C − F_ = 8.1 Hz), 119.0 (d, ^3^*J*_C − F_ = 7.8 Hz), 119.4 (^2^*J*_C − F_ = 23.3 Hz), 124.6, 124.7, 125.9, 153.0, 153.2, 154.6, 155.5 (d, ^2^*J*_C − F_ = 234.5 Hz), 168.1. HRMS [M − H]^−^: calculated 271.0524; found 271.0525.

*2-(5-Chloro-2-hydroxyphenyl)-1H-benzo[d]imidazole-5-carboxylic acid (***7d***).* White solid, m.p. > 300 °C, 72% yield. ^1^H NMR (300 MHz, DMSO-d_6_), δ (ppm): 7.10 (d, 1 H, *J* = 6.0 Hz), 7.44 (d, 1 H, *J* = 6.0 Hz), 7.73–7.75 (m, 1 H), 7.90 (d, 1 H, *J* = 6.0 Hz), 8.19 (s, 1 H), 8.24 (broad s, 1 H), 12.86 (broad s, 1 H, COOH). ^13^C NMR (150 MHz, DMSO-d_6_) δ (ppm): 119.6, 123.4, 124.8, 125.9, 126.5, 132.2, 152.7, 157.1, 168.0. HRMS [M − H]^−^: calculated 287.0229; found 287.0228.

*2-(5-Nitro-2-hydroxyphenyl)-1H-benzo[d]imidazole-5-carboxylic acid (***7e***).* White solid, m.p. > 300 °C, 78% yield. ^1^H NMR (600 MHz, DMSO-d_6_) δ (ppm): 7.06–7.11 (m, 1 H), 7.25–7.32 (m, 1 H), 7.73–7.76 (m, 1 H), 7.89–7.96 (m, 2 H), 8.24 (broad s, 1 H), 12.78 (broad s, 1 H). ^13^C NMR (150 MHz, DMSO-d_6_) δ (ppm): 113.3, 114.4, 118.9, 122.1, 123.4, 126.9, 127.5, 128.1, 129.1, 129.5, 152.7, 169.2. HRMS [M − H]^−^: calculated 298.0469; found 298.0468.

*2-(4-Methoxy-2-hydroxyphenyl)-1H-benzo[d]imidazole-5-carboxylic acid (***7f***).* White solid, m.p. > 300 °C, 67% yield. ^1^H NMR (600 MHz, DMSO-d_6_), δ (ppm): 3.79 (s, 3 H), 6.59–6.63 (m, 2 H) 7.66 (d, *J* = 9.0 Hz, 1 H), 7.86 (d, *J* = 9.0 Hz, 1 H), 8.10 (d, *J* = 9.0 Hz, 1 H), 8.18 (s, 1 H), 12.96 (broad s, 1 H). ^13^C NMR (150 MHz, DMSO-d_6_) δ (ppm): 55.9, 101.9, 105.8, 107.2, 124.4, 125.3, 128.6, 154.4, 160.4, 163.1, 168.1. HRMS [M − H]^−^: calculated 283.0724; found 283.0728.

*2-(5-Methoxy-2-hydroxyphenyl)-1H-benzo[d]imidazole-5-carboxylic acid (***7g***).* White solid, m.p. > 300 °C, 79% yield. ^1^H NMR (600 MHz, DMSO-d_6_), δ (ppm): 3.33 (s, 3 H), 6.64 (d, *J* = 9.0 Hz, 1 H), 6.74 (d, *J* = 9.0 Hz, 1 H), 7.19 (s, 1 H), 7.90–7.92 (m, 1 H), 8.09–8.11 (m, 1 H), 8.27 (s, 1 H), 10.2 (broad s, 1 H). ^13^C NMR (150 MHz, DMSO-d_6_) δ (ppm): 56.2, 110.7, 112.7, 118.6, 119.8, 124.3, 125.7, 150.2, 152.5, 154.1, 168.1. HRMS [M − H]^−^: calculated 283.0724; found 283.0725.

*2-(2,4-Dihydroxyphenyl)-1H-benzo[d]imidazole-5-carboxylic acid (***7h***).* White solid, m.p. > 300 °C, 94% yield. ^1^H NMR (300 MHz, DMSO-d_6_), δ (ppm): 6.43 (s, 1 H) 6.47 (d, *J* = 6.0 Hz, 1 H), 7.65 (d, *J* = 6.0 Hz, 1 H), 7.86 (d, *J* = 9.0 Hz, 1 H), 7.89 (d, *J* = 9.0 Hz, 1 H), 8.15 (s, 1 H), 12.96 (broad s, 1 H). ^13^C NMR (150 MHz, DMSO-d_6_) δ (ppm): 103.5, 104.7, 108.3, 113.1, 113.2, 115.8, 120.8, 125.2, 128.4, 140.5, 155.9, 160.4, 161.7, 168.5. HRMS [M − H]^−^: calculated 269.0568; found 269.0564.

### 3.7. Synthesis and Characterization Data of N-(2-(4-benzylpiperazin-1-yl)ethyl)-2-(2-hydroxy-phenyl)-1H-benzo[d]imidazole-5-carboxamide (PZ1) (**8**)

Table 3, entry 1: a mixture of **4** (0.5 mmol), **7a** (0.5 mmol), *N*-hydroxysuccinimide (0.5 mmol) and *N,N*′-dicyclohexylcarbodiimide (0.5 mmol) in anhydrous DMF (3 mL) was stirred at room temperature for 60 h under a nitrogen atmosphere. After this time, the resulting mixture was filtered on a Büchner funnel, diluted with AcOEt (10 mL), and washed with brine (10 mL). The organic phase was dried over anhydrous Na_2_SO_4_ and concentrated under reduced pressure. The crude product was purified by flash-chromatography (silica gel; eluent: CH_2_Cl_2_/MeOH 98:2:) to give **8** as a yellow solid (21% yield).

Table 3, entry 3: compounds **4** (0.5 mmol), **7a** (0.5 mmol), *N*-hydroxysuccinimide (0.5 mmol) and *N,N*′-dicyclohexylcarbodiimide (0.5 mmol) were progressively dissolved in a ChCl/PG eutectic mixture (1.0 g) under magnetic stirring, and the resulting mixture was then heated at 60 °C for 60 h. After this time, the reaction mixture was cooled to room temperature and 5 mL of H_2_O were added. The resulting aqueous suspension was then extracted with AcOEt (3 × 10 mL). The combined organic phases were washed with brine (10 mL), dried over anhydrous Na_2_SO_4_ and concentrated under reduced pressure. The crude product was purified by flash-chromatography (silica gel; eluent: AcOEt/MeOH 98:2) to give **8** as a yellow solid (30% yield).

*N-(2-(4-Benzylpiperazin-1-yl)ethyl)-2-(2-hydroxy-phenyl)-1H-benzo[d]imidazole-5-carboxamide (***PZ1***)* (**8***)*. Yellow solid, m.p.= 249–252 °C. ^1^H NMR (400 MHz, DMSO-d_6_), δ (ppm): 2.34–2.47 (m, 10 H, 8 piperazine and 2 NH_2_–CH_2_CH_2_N), 3.36–3.41 (m, 4 H, 2 NCH_2_ Ph and 2 NH_2_–CH_2_CH_2_N), 6.99–7.09 (m, 2 H), 7.19–7.34 (m, 4 H), 7.39–7.44 (m, 1 H), 7.65–7.72 (m, 1 H), 7.76–7.83 (m, 1 H), 8.05–7.11 (m, 1 H), 8.13–8.15 (m, 1 H), 8.41 (t, *J* = 5.3 Hz, 1 H). ^13^C NMR (100.5 MHz, DMSO-d_6_), δ (ppm): 37.0, 52.7, 52.9, 57.1, 62.2, 112.5, 117.3, 119.3, 122.4, 126.6, 126.9, 128.2, 128.6, 129.4, 132.2, 138.3, 153.3, 158.1, 166.4. HRMS [M + H]^+^: calculated 456.2394; found 456.2375.

## 4. Conclusions

In this paper, we have described an efficient condensation-mediated synthesis of 2-hydroxyphenylbenzimidazole derivatives and the whole synthesis of the hit, Donepezil-like compound PZ1, in selected DESs as environmentally responsible, safe, and nonconventional reaction media. Compared to VOCs, the synthesis of 2-hydroxyphenylbenzimidazoles in ChCl/gly takes place in better yields, shorter reaction time (30 min vs. 12 h) and under milder conditions (50 vs. 100 °C), and provides a way of easy functionalization of the phenolic moiety which may have implication on some important biological properties of these nuclei, such as the effective chelation of heavy metals. Moreover, these adducts were found to precipitate from the eutectic mixture after adding water during the work-up procedure, and thus they could easily be isolated by simple filtration. Further investigation into the application of these ligands in the therapy of Alzheimer’s disease are underway in our laboratory and results will be reported in due course.

## Figures and Tables

**Table 1:**
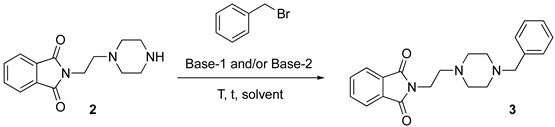
Optimization of the benzylation reaction for the synthesis of **3**. ^a^

Entry	Base-1 (Equiv)	Base-2 (Equiv)	BnBr (Equiv)	T (°C)	T (h)	Solvent	3, Yield (%) ^b^
1	TEA (1.4)	K_2_CO_3_ (6.6)	2.3	50	3	CH_3_CN	64 ^c^
2	KOH (1.1)	-	1.0	50	24	ChCl/gly	NR ^d^
3	KOH (1.1)	-	1.0	50	24	ChCl/urea	NR ^d^
4	*t*-BuOK (1.1)	-	1.0	50	24	ChCl/gly	24
5	*t*-BuOK (1.1)	-	1.0	50	24	ChCl/urea	10
6	*t*-BuOK (1.1)	-	1.0	50	24	ChCl/PG	20
7	*t*-BuOK (1.1)	-	1.0	100	24	d-fructose/ChCl	8
8	*t*-BuOK (1.1)	-	1.0	100	24	d-fructose /urea	8
9	*t*-BuOK (1.1)	-	1.0	50	24	ChCl/gly	24
10	*t*-BuOK (1.1)	-	1.0	50	24	ChCl/urea	10
11	TEA (1.4)	K_2_CO_3_ (6.6)	2.3	50	24	ChCl/gly	NR ^d^
12	K_2_CO_3_ (2.0)	-	1.0	50	24	ChCl/gly	NR ^d^
13	TEA (2.0)	-	1.0	50	24	ChCl/gly	26
14	TEA (2.0)	-	1.0	50	24	ChCl/PG	44
15	TEA (2.0)	-	2.0	50	24	ChCl/PG	64 ^c^
16	TEA (2.0)	-	2.0	50	24	ChCl/gly	61 ^c^
17	TEA (2.0)	-	1.0	50	24	DL-menthol/LA	NR ^d^
18	TEA (2.0)	-	2.0	50	24	Bu_4_NBr/gly	44 ^c^
19	TEA (2.0)	-	2.0	50	24	Bu_4_NCl/gly	68 ^c^

^a^ Reaction conditions in deep eutectic solvent (DES): 1.0 g DES per 0.5 mmol of **2**; DES: ChCl/propylene glycol (PG) (1:3, mol mol^−1^); ChCl/gly (1:2 mol mol^−1^); ChCl/urea (1:2 mol mol^−1^); d-fructose/ChCl (2:1 mol mol^−1^); d-fructose/urea (3:2 *w*/*w*); dl-menthol/L-lactic acid (LA) (1:2 mol mol^−1^); Bu_4_NBr/gly (1:4 mol mol^−1^); Bu_4_NCl/gly (1:4 mol mol^−1^). ^b^ Calculated via ^1^H-NMR analysis of the crude reaction mixture using an internal standard technique (NMR internal standard: dibromomethane). ^c^ The yields reported are for isolated products. ^d^ NR = no reaction.

**Table 2:**
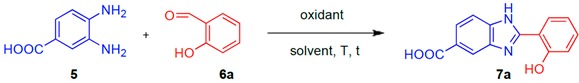
Optimization of the synthesis of 2-hydroxyphenylbenzimidazole **7a**. ^a^

Entry	T (°C)	T (h)	Oxidant	Solvent	7a, Yield (%) ^b^
1	100	0.5	Na_2_S_2_O_5_	ChCl/urea	66
2	100	0.5	Na_2_S_2_O_5_	ChCl/gly	80
3	100	0.5	Na_2_S_2_O_5_	ChCl/LA	76
4	50	0.5	Na_2_S_2_O_5_	ChCl/gly	84
5	25	24	Na_2_S_2_O_5_	ChCl/gly	36
6	50	0.5	urea-H_2_O_2_	ChCl/gly	17 ^c^
7	50	0.5	- ^d^	ChCl/gly	29 ^c^
8	100	12	Na_2_S_2_O_5_	DMA	67

^a^ Reaction conditions in DES: 1.0 g DES per 0.5 mmol of **5**, 0.5 mmol of **6a** and 0.7 mmol of oxidant; DES: ChCl/gly (1:2 mol mol^−1^); ChCl/urea (1:2 mol mol^−1^); ChCl/LA (1:2 mol mol^−1^); ^b^ The yields reported are for isolated products. ^c^ Calculated via ^1^H-NMR analysis of the crude reaction mixture using an internal standard technique (NMR internal standard: dibromomethane). ^d^ Under air.

**Table 3:**
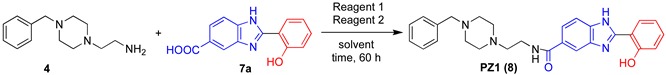
Optimization of the synthesis of PZ1 (**8**). ^a^

Entry	Reagent 1 (Equiv)	Reagent 2 (Equiv)	T (°C)	Solvent	8, Yield (%) ^b^
1	NHS (1)	DCC (1)	25	DMF	21
2	NHS (1)	DCC (1)	60	ChCl/gly	16
3	NHS (1)	DCC (1)	60	ChCl/PG	30
4	NHS (1)	DCC (1)	60	ChCl/urea	13
5	NHS (1)	DCC (1)	60	menthol/LA	NR ^c^
6	NHS (1)	DCC (1)	60	d-fructose/urea	NR ^c^
7	NHS (1)	DCC (1)	60	Bu_4_NBr/gly	7 ^d^
8	NHS (1)	DCC (1)	60	Bu_4_NCl/gly	19 ^d^
9	NHS (1)	-	60	ChCl/PG	<5 ^d^
10	-	DCC (1)	60	ChCl/PG	NR ^c^

^a^ Reaction conditions in DES: 1.0 g DES per 0.5 mmol of **4**; DES: ChCl/gly (1:2 mol mol^−1^); ChCl/urea (1:2 mol mol^−1^); ChCl/PG (1:3 mol mol^−1^); d-fructose/urea (3:2 *w*/*w*); dl-menthol/LA (1:2 mol mol^−1^); Bu_4_NBr/gly (1:4 mol mol^−1^); Bu_4_NCl/gly (1:4 mol mol^−1^). ^b^ The yields reported are for isolated products. ^c^ No reaction. ^d^ Calculated via ^1^H-NMR analysis of the crude reaction mixture using an internal standard technique (NMR internal standard: dibromomethane).
